# Reversal of metformin’s anti-proliferative effect in fission yeast *efr3* and *dnm1* (DRP1) mutants with elongated mitochondria

**DOI:** 10.1038/s44324-024-00048-9

**Published:** 2025-02-21

**Authors:** Ari Gillespie, Anne-Sophie Mehdorn, Tiffany Q. Lim, Tingting Wang, Bridget A. Mooney, Ashley J. Ovens, Ayla Orang, Jonathan S. Oakhill, Michael Z. Michael, Janni Petersen

**Affiliations:** 1 Flinders Health and Medical Research Institute, Flinders Centre for Innovation in Cancer, Flinders University, Adelaide, SA 5042 Australia; 2 Metabolic Signalling Laboratory, St Vincent’s Institute of Medical Research, Fitzroy, Victoria, 3065 Australia; 3 Department of Medicine, University of Melbourne, Parkville, Victoria, 3010 Australia; 4 Flinders Centre for Innovation in Cancer, Dept. Gastroenterology and Hepatology, Flinders Medical Centre, Bedford Park, Adelaide, SA 5042 Australia; 5 South Australia Health and Medical Research Institute, Adelaide, South Australia Australia; 6 Present Address: Department of General, Abdominal, Thoracic, Transplantation and Pediatric Surgery, University Hospital Schleswig-Holstein, Campus Kiel, Arnold-Heller-Straße 3, 24105 Kiel, Germany

**Keywords:** Cancer, Cell biology

## Abstract

Metformin is a well-tolerated drug frequently prescribed for managing type 2 diabetes. Extended metformin use has been linked to a significant decrease in cancer incidence across both diabetic and non-diabetic populations. Here we investigate the anti-proliferative effects of metformin on fission yeast *S. pombe*. Our findings demonstrate that metformin’s inhibitory impact on cell proliferation is effective in the absence of AMP-activated protein kinase (AMPK). Using an unbiased genetic screen we identified the plasma membrane signalling scaffold Efr3, critical for phosphatidylinositol signalling and the generation of PI4Ps, as a key determinant of resistance to the anti-proliferative effect of metformin. Deletion of *efr3* resulted in both AMPK-dependent and AMPK-independent resistance to metformin. We show that Efr3 does not influence cell proliferation by controlling Ras1 activity or its cellular localization in yeast. We observe that *dnm1* (DRP1) mutants with elongated mitochondria are also resistant to the anti-proliferative effect of metformin and that metformin treatment promotes mitochondrial fusion. Metabolic measurements after prolonged metformin exposure demonstrated a reduction in respiration in both wild type and the *efr3* deletion, however, that reduction is less pronounced in the *efr3* deletion, which also contained elongated mitochondria. It is likely that mitochondrial fusion enhances yeast fitness in response to metformin exposure. Together we provide a new perspective on the cellular response to metformin.

## Introduction

Metformin, a highly tolerable oral medication, is commonly used in the treatment of type 2 diabetes mellitus. It operates through a variety of mechanisms, one of which includes the lowering of blood glucose levels^1,2^. Importantly, the use of metformin has been linked to a significant decrease in cancer incidence across both diabetic and non-diabetic populations. Moreover, meta-analyses have indicated a favourable correlation between metformin and improved survival rates in a variety of cancer types^3–8^.

At the molecular level, the mechanisms of action appear to vary depending on the dosage and duration of metformin treatment. The initial mechanism proposed that metformin lowers cellular energy levels through the inhibition of Complex 1 of the mitochondrial electron transport chain^9^. This inhibition blocks the conversion of adenosine diphosphate (ADP) into adenosine triphosphate (ATP), which subsequently triggers an increase in AMP/ATP and ADP/ATP ratios, eventually contributing to the activation of the AMP-activated protein kinase (AMPK). Recently, further evidence for this mechanism was provided by a combined analysis of structure and enzyme kinetics, revealing the binding site of metformin on respiratory complex I^10^. Furthermore, targets on the lysosome surface, when lower metformin concentrations are used, suggest alternative modes of action, also regulated by the AMPK pathway^11^. In addition to these effects on AMPK, a variety of actions that are independent of AMPK have been reported. These include miRNA modulation, generation of reactive oxygen species (ROS), suppression of angiogenesis, modulation of mammalian target of rapamycin (mTOR) pathways, regulation of inflammatory processes, and enhancement of glucose uptake^12,13^.

Whether the modifications of AMPK and mTOR activities following cell exposure to metformin^13,14^ are key mechanisms behind its anti-proliferative effect is not yet fully understood and has been a subject of debate^15,16^. Coordinating signalling through these primary nutrient and energy-sensing pathways plays a crucial role in the regulation of cell growth and proliferation^17^. AMPK functions as a heterotrimeric kinase, composed of α, β, and γ subunits, with the catalytic domain residing in the α1 and α2 subunits. As mentioned, AMPK is activated when intracellular ratios of AMP/ATP and ADP/ATP increase, typically during periods of nutritional stress^18^. Once activated, AMPK constrains energy-intensive anabolic pathways, in part by inhibiting mTOR. Additionally, AMPK signalling stimulates catabolic pathways such as autophagy, facilitating the recycling of nutrients and the restoration of cellular equilibrium^17^. On the other hand, mTOR, functioning as the catalytic subunit, forms an integral part of two unique multi-protein complexes. The complexes are distinguished by specific regulatory binding partners, with Raptor defining mTOR complex 1 (mTORC1), and Rictor defining mTOR complex 2 (mTORC2). In nutrient-replete conditions, mTORC1 drives energy-demanding anabolic processes, thereby supporting cell growth and proliferation^19^, whereas mTORC2 is required for cell survival.

In this study, we used the *Schizosaccharomyces pombe* fission yeast model organism, in which all key components of AMPK and mTOR are conserved, to investigate whether modification of AMPK signalling is a key mechanism behind the anti-proliferative effect of metformin.

Here we show that the metformin induced block to cell proliferation is operative in the absence of AMPK. We identify Efr3 as a key determinant of resistance to the anti-proliferative effect of metformin, with deletion of *efr3* resulting in both AMPK-dependent and AMPK-independent resistance. We show that Efr3 regulates cell proliferation independently of Ras1 signalling. We also observe that *dnm1* (DRP1) mutants with elongated mitochondria are resistant to metformin and that metformin treatment promotes mitochondrial fusion, likely enhancing cell fitness.

## Results

### The anti-proliferative effect of metformin is reversed in fission yeast efr3 mutants

**Fig. 1:**
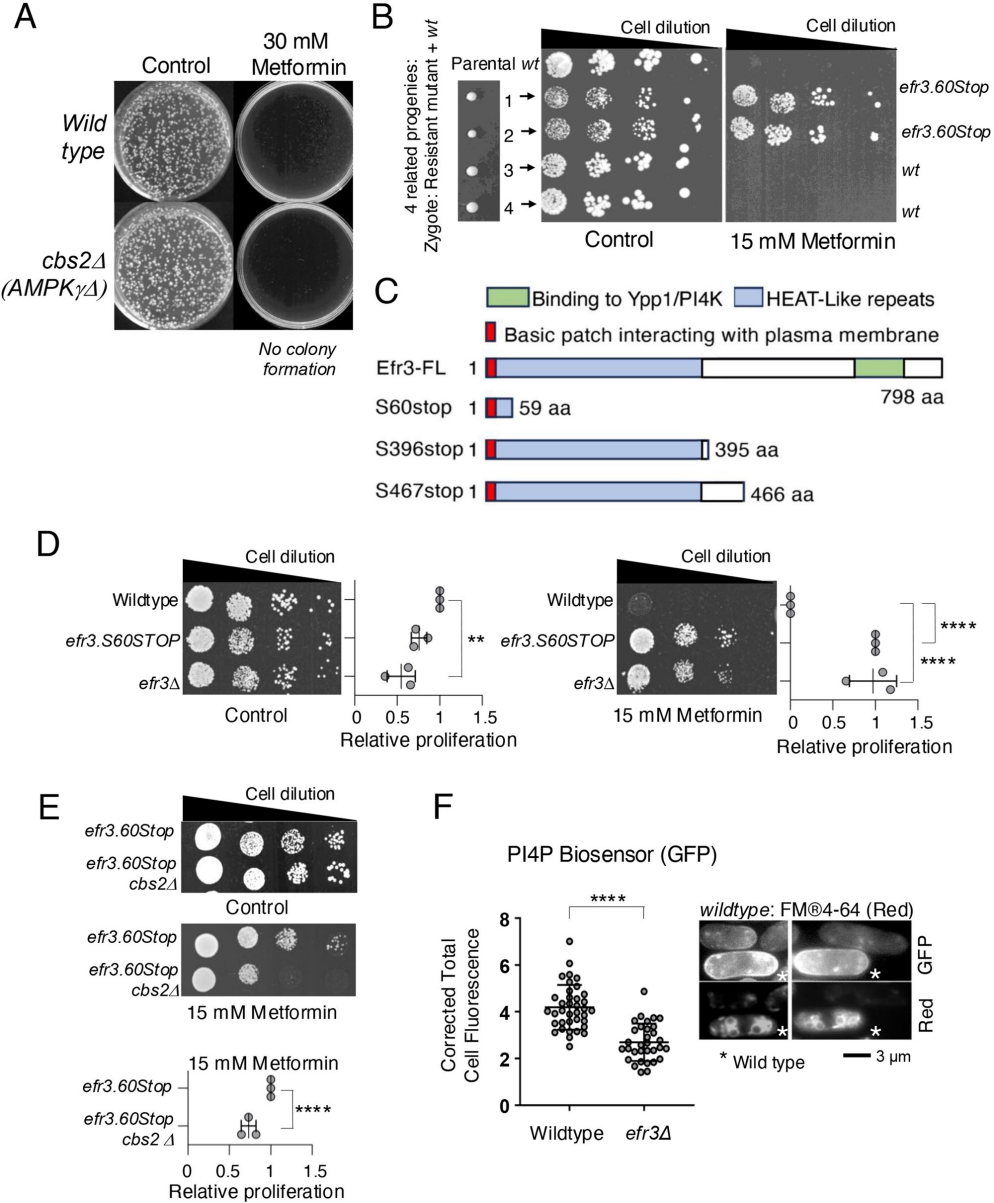
The anti-proliferative effect of metformin is reversed in fission yeast efr3 mutants. **A** Colony forming assay demonstrates that the anti-proliferative impact of metformin is independent of AMPK. **B** Meiosis and sporulation were promoted in a zygote formed from a metformin-resistant mutant and wild-type fission yeast. A growth assay of 4 related progenies demonstrated 2:2 mendelian segregation of resistance to the antiproliferative effect of metformin. Sanger sequencing identified a stop codon at amino acid position 60 in *efr3*. **C** Sanger sequencing of 3 independent metformin-resistant mutants, all identified premature stop codons in the *efr3* gene. The carboxy terminus interacts with Ypp1/and the PI4 kinase Stt4^63^. **D**, **E** Growth characteristics of indicated strains on rich YES media with or without 15 mM metformin. The quantification of three independent biological repeats is shown. **F** Live cell imaging of the PI4P biosensor (GFP) in indicated strains. To differentiate between the two cell types the two strains were mixed at a ratio 1:1 immediately prior to imaging. Wild-type cells were initially stained with FM®4-64 (accumulates in the vacuoles, indicated by a star) for 45 min, before they were mixed with the *efr3* deletion for immediate imaging. The relative fluorescence intensity of the PI4P biosensor (GFP) in all cells was quantified as: overall fluorescence intensity relative to image background. FM®4-64 staining does not affect PI4P biosensor (GFP) fluorescence. Scale bar = 3 μm. Statistics are calculated from images of one experiment. Error bars represent mean +/- Standard deviation. Representative images are shown. Similar results were obtained for three independent biological repeats.

To gain deeper insights into the mechanisms underlying metformin’s anti-proliferative effects, we adopted an unbiased genetic approach to identify metformin-regulated signalling. First, we established an appropriate metformin concentration to use in this genetic screen. It was recently demonstrated that the addition of 25 mM metformin to a liquid fission yeast culture initially reduced cell proliferation before prolonging the survival and lifespan of cells in the stationary phase^20^. Our initial proliferation assays, which assessed the ability of non-starved individual cells to form colonies, revealed that both wild-type fission yeast and cells lacking AMPK activity (due to the deletion of the AMPK gamma subunit) stopped cell proliferation when plated onto agar plates with 30 mM metformin (Fig. 1A). This observation suggests that the inhibitory impact of 30 mM metformin on cell proliferation remains effective in the absence of AMPK. Therefore, fission yeast emerges as a model system in which AMPK-dependent and independent effects of metformin can be explored. To induce random mutagenesis of the genome, we subjected both the wild type and an AMPK deletion mutant to UV radiation. Subsequently, we selected mutants resistant to Metformin, employing a strategy we had successfully utilized previously to validate the fission yeast TOR kinase as the primary target of Tor inhibitor 1 (Torin1)^21^. Specifically, the screen identified mutants that gained the ability to form colonies on rich media YES plates containing 30 mM metformin. Six mutants were isolated from the wild-type strain and one resistant mutant from the AMPK deletion strain. Backcrosses of these mutants to wild-type cells showed 2:2 Mendelian segregation (Fig. 1B) for all mutants. Further, crosses of the mutants to each other revealed that all belonged to the same complementation group. Genome-wide sequencing of 3 of these mutants identified single point mutations in the *efr3* gene, these all introduced premature stop codons and hence truncation of the Efr3 carboxy terminus (Fig. 1C). The *efr3.S60Stop* mutation generated the largest truncation, resulting in most of the protein being deleted. An *efr3* deletion strain confirmed that the lack of *efr3* function confers resistance to Metformin (Fig. 1D). Interestingly, the ability of *efr3.S60Stop* to proliferate in the presence of metformin was partly dependent on AMPK, as the *efr3.S60Stop cbs2.Δ* (deletion of AMPK gamma subunit) double mutant showed reduced growth on metformin plates (Fig. 1E). This suggests that the *efr3* deletion confers both AMPK-dependent and AMPK-independent metformin resistance.

Efr3 is a highly conserved protein that binds to the plasma membrane at its amino terminus, with its carboxy terminus functioning as a scaffold for protein-protein interactions. Specifically, it forms a complex with Ypp1 (TTC7A/B), facilitating the binding of Stt4 PI4K (PI4KIIIa) to the plasma membrane. This interaction is critical for phosphatidylinositol signalling and the generation of PI4Ps at the plasma membrane^22^. Functionally, PI4Ps at the plasma membrane act as precursors for the synthesis of PIP2 and PIP3, essential signalling lipids^23^. To validate Efr3’s role in PI4P generation, we employed a biomarker specific to PI4Ps, the P4C domain from *Legionella pneumophila* SidC domain fused to GFP^22^ this marker selectively binds to PI4Ps. Live-cell imaging of early exponential cultures confirmed a significant reduction, by approximately 50%, in total PI4Ps within the *efr3* deletion cells^22^, known to be enriched on the Golgi apparatus and the plasma membrane^24^ (Fig. 1F).

### Fission yeast Efr3 functions independently of Ras1 signalling

**Fig. 2:**
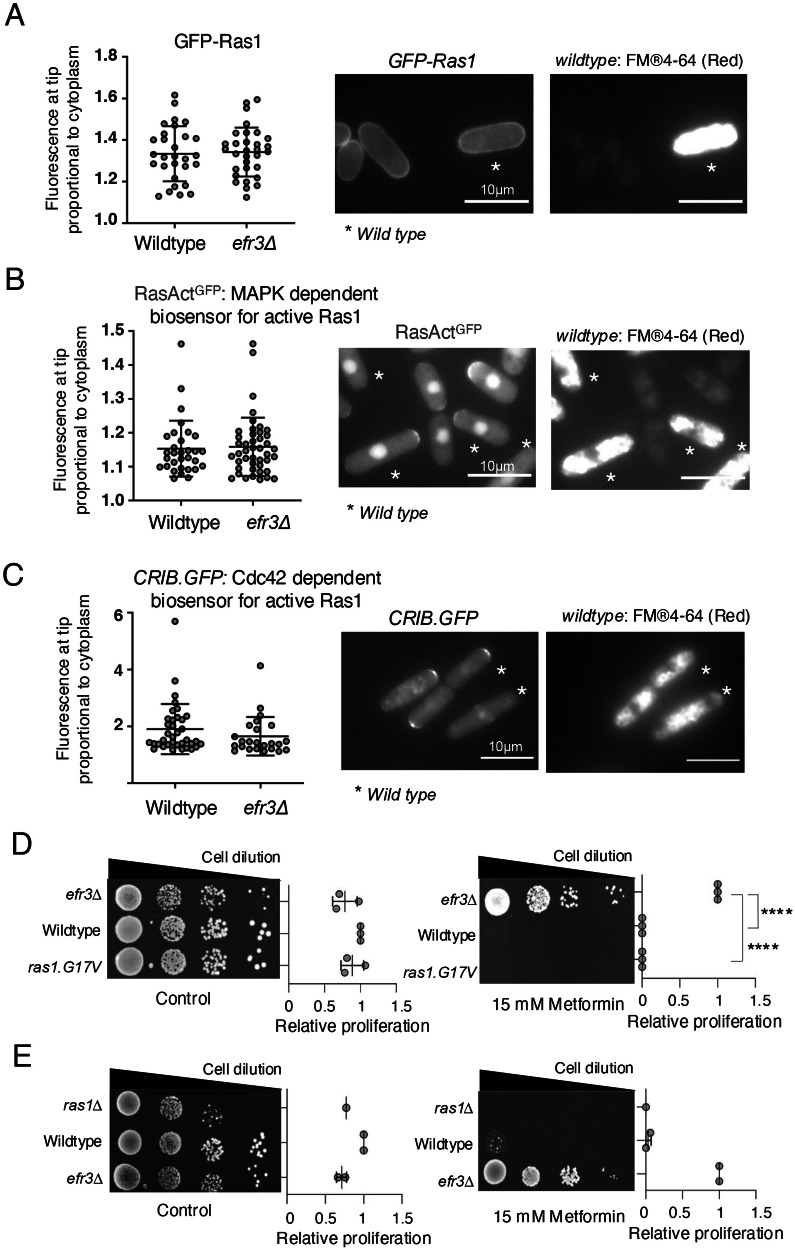
Efr3 does not control cell proliferation through Ras1 activity or localization. **A–C** Live cell imaging of the Ras1 localisation and biosensors of Ras1 activity in indicated strains. To differentiate between the two cell types (the two strains were mixed at a ratio 1:1 immediately prior to imaging) wild type cells were initially stained with FM®4-64 (accumulates in the vacuoles, indicated by a star) for 45 min, before they were mixed with the *efr3* deletion for immediate imaging. The relative fluorescence intensity in all cells was quantified as: overall fluorescence intensity at cell tips relative to cytoplasmic background. Scale bar = 3 μm. Stats are calculated from images of one experiment. Representative images are shown. Similar results were obtained for three independent biological repeats. Error bars represent mean +/- Standard deviation. **D**, **E** Growth characteristics of indicated strains on rich YES media with or without 15 mM metformin. The quantification of three independent biological repeats is shown.

Recently, a novel signalling mechanism was discovered for human EFR3A and EFR3B^25^. This mechanism demonstrated a role in facilitating the localization and activation of KRAS to the plasma membrane. Fission yeast expresses a single RAS homolog, Ras1^26^, which, although not essential for mitotic growth, influences the rate of proliferation, leading to reduced growth of the *ras1* deletion. Fission yeast Ras1 localizes to the plasma membrane^27^. To investigate the dependency of Efr3 on Ras1 localization in fission yeast, we incorporated GFP-Ras1 into the *efr3* deletion mutant. To observe Ras1 localisation, cultures of wild-type and *efr3.Δ* cells expressing GFP-Ras1 were grown to early exponential phase, with the wild-type cells serving as a reference. Wild-type cells were subjected to a 30-minute staining period using SynaptoRed, a dye known to accumulate in vacuoles (Fig. 2A). A 1:1 mixture of the two strains was prepared just before imaging to facilitate the distinction between the strains. No significant difference in GFP-Ras1 localisation was observed in the *efr3* deletion strain (Fig. 2A), the same result was obtained when the *efr3* deletion was stained with SynaptoRed (data not shown). In agreement with this observation, two distinct biosensors for Ras1 activity, namely RasActGFP^27^ and CRIB-GFP^28^, each relying on downstream effectors MAPK and Cdc42 activity, respectively, demonstrated no apparent difference in *efr3* deletions when compared to wild-type cells (Fig. 2B, C), the same results were obtained when the *efr3* deletion was stained with SynaptoRed (data not shown). Furthermore, neither constitutively active *ras1.G17V* nor *ras1* deletion conferred resistance to metformin (Fig. 2D, E). Together, these observations suggest that Efr3 is not required for Ras1 localisation and activity in fission yeast, and the ability of *efr3* mutants to proliferate in the presence of metformin (Fig. 1) is likely unrelated to Ras1 signalling.

### The efr3 deletion fails to proliferate when complex III of the respiratory chain is inhibited

**Fig. 3:**
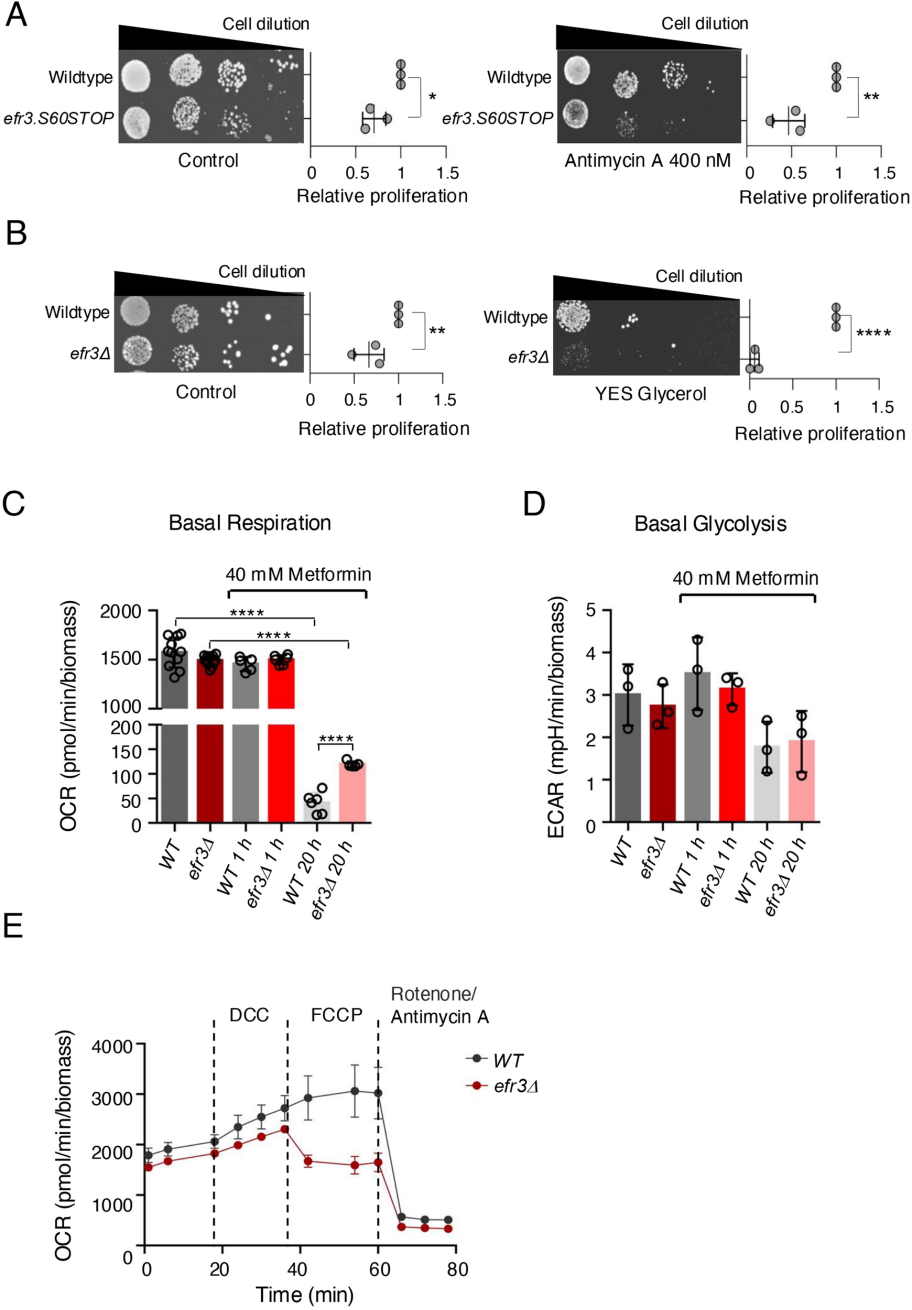
Basal respiration is higher in *efr3* deletion compared to wild type following prolonged metformin treatment. **A**, **B** Growth characteristics of indicated strains. The quantification of three independent biological repeats is shown. **A** Growth on rich YES media with or without 400 nM Antimycin A. **B** Growth on rich YES media with or without glucose vs glycerol. **C**, **D** Basal Metabolic measurements of the *efr3* deletion and wild type cells with and without the addition of 40 mM metformin. Similar results were obtained for three independent biological repeats. Error bars represent mean +/- Standard deviation. **E** Mitochondria stress tests measuring oxygen consumption rates (OCR) of wild type and the efr3 deletion. 8.25 µM DCC, 21.5 µM FCCP, 500 nm Rotenone and 900 nM Antimycin A were added at indicated times. The efr3 deletion is sensitive to the mitochondrial uncoupler FCCP. Error bars represent mean +/- Standard deviation.

Given that metformin in part functions by inhibiting the mitochondrial respiratory chain through its binding to Complex I^9,10^, we examined the sensitivity of *efr3* deletion to an alternative, potent inhibitor of the electron transport chain—Antimycin A, which inhibits complex III of the respiratory chain leading to a decrease in ATP production^29^. Cells lacking functional *efr3* were sensitive to Antimycin A (Fig. 3A) rather than resistant as seen for metformin (Fig. 1). We next assessed cell proliferation on glycerol as the sole carbon source to induce mitochondrial stress. Fission yeast can proliferate slowly on glycerol but lacks glycerol kinase so instead assimilate glycerol through generation of dihydroxyacetone (DHA) by glycerol dehydrogenase (Gld1)^30^. DHA exposure can induce mitochondrial stress and supress TOR signalling^31,32^, which in part may explain why fission yeast grow very slowly on glycerol. In contrast to wild type, the *efr3* deletion were unable to proliferate on glycerol as sole carbon source (Fig. 3B). Together, these observations suggest that mitochondrial function may be altered in *efr3* deletion strains.

### Metformin reduces respiration to a greater extent in wild-type cells compared to efr3 deletion cells

To determine whether metformin impacts the mitochondrial respiratory chain in fission yeast, and if so, whether this effect differs in *efr3* deletion mutants, we performed metabolic measurements. First, to establish an appropriate metformin concentration for use in liquid minimal cultures, we assessed cell proliferation in a range of metformin concentrations (Figure S1A, B). Metformin was added to early-exponential cultures (1.5 ×10^6 cells/ml) grown in liquid minimal media (EMM2). Acute treatment with 40 mM metformin slightly reduced the growth rates of wild-type cells but not efr3 deletion mutants (Figure S1A). After 24 hours of treatment with 40 mM metformin, both wild-type and efr3 deletion cells reached the stationary phase with the same biomass, indicating that metformin is not cytotoxic at this concentration. In cultures treated with 50, 60 and 70 mM metformin the *efr3* deletion reached a significantly higher biomass when compared to wild-type cells (Figure S1B). We also demonstrated that the *efr3* deletion is sensitive to Antimycin A at a lower concentration of 10 nM (Figure S1B). To perform real-time metabolic measurements, we used the Agilent Seahorse XF Analyzer. Interestingly, basal respiration and glycolysis levels were indistinguishable between control early-exponential wild-type cultures and *efr3* deletion cultures, both before and after 1 hour of treatment with 40 mM metformin (Fig. 3C, D). In contrast, 20 hours after the addition of 40 mM metformin, respiration, but not glycolysis, was strongly reduced in both wild-type and *efr3* deletion cultures. However, respiration was significantly higher in the *efr3* deletion mutant (Fig. 3C). These results demonstrate that metformin inhibits respiration in fission yeast, but not to the same extent in *efr3* deletion mutants compared to wild-type cells.

### The efr3 deletion is sensitive to the mitochondria uncoupler FCCP

We next performed a mitochondria stress and glycolysis stress test, to get insight into respiratory capacity and glycolytic capacity of wild type and the *efr3* deletion. Both tests rely on oligomycin or, alternatively, N,N-Dicyclohexylcarbodiimide (DCC) to inhibit ATP synthase in the mitochondria. Unfortunately, neither oligomycin nor DCC had an inhibitory effect on respiration in fission yeast (Fig. 3E) and data not shown, as reported previously in budding yeast^33^. We were therefore unable to perform the full mitochondria stress and glycolysis stress tests. However, we found that the *efr3* deletion was sensitive to the mitochondrial uncoupler Carbonyl cyanide-4 (trifluoromethoxy) phenylhydrazone (FCCP) (Fig. 3E), which disrupts the proton gradient across the mitochondrial membrane to induce maximal respiration. An inhibitory effect of FCCP on the uptake of substrates into the mitochondrial matrix has been suggested previously^34,35^. Whether the availability of substrates and the sensitivity of *efr3* deletions to both FCCP and Antimycin A (Fig. 3B, S1B) are related is unknown and requires further studies. Investigations of glycolysis demonstrated that it was reduced and indistinguishable between wild type and the *efr3* deletion after 1 hour of glucose starvation both with and without the addition of 40 mM metformin (Figure S1C). Supplementation of glucose restored glycolysis in all cultures, however both wild type and the *efr3* deletion treated with 40 mM metformin for 1 hour demonstrated significantly higher levels of glycolysis (Figure S1C). Hence, acute treatment of metformin appears to enhance the glycolytic capacity in both strains: interestingly, this effect is lost after 20 hours of metformin treatment. It has been reported previously that metformin can upregulate glycolysis and key genes involved in glycolysis^36^. Together our metabolic measurements show that metformin’s impact on glycolysis is indistinguishable between wild type and the *efr3* deletion.

### Dnm1 mutants with elongated mitochondria are resistant to metformin

**Fig. 4:**
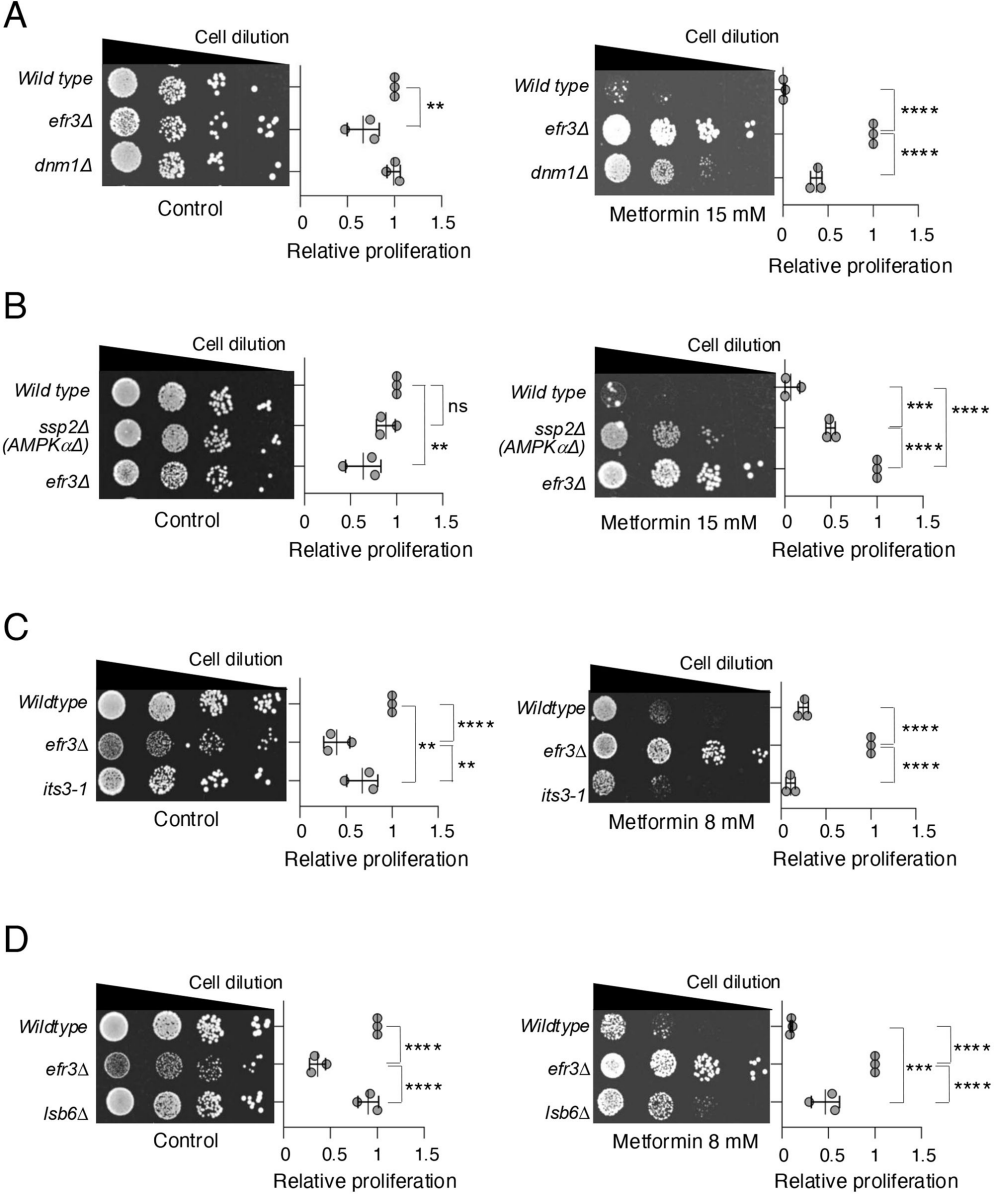
The anti-proliferative effect of metformin is reversed in *dnm1* (Drp1 homolog), *ssp2* (AMPKα homolog) and *lsb6* (PI4-kinase) deletions. **A–D** Growth characteristics of indicated strains. The quantification of three independent biological repeats is shown. Error bars represent mean +/- Standard deviation. **A**, **B** Growth on rich YES media with or without 15 mM metformin. **C**, **D** Growth on rich YES media with or without 8 mM metformin.

Our metabolic measurements indicate that basal respiration is elevated in the *efr3* deletion when compared to wild type following prolonged exposure to metformin (Fig. 3C). Mitochondrial elongation and tubulation have been linked to enhanced mitochondrial function^37^ and metformin can induce mitochondrial fission in human cells, subject to the cell type and environment^38–42^. Therefore, we examined whether mutants defective in mitochondrial fission, thus having elongated and tubular mitochondria networks, show altered response to the anti-proliferative effect of metformin. Remarkably, mutants in the dynamin-related GTPase Dnm1 (Drp1 in human cells)^43^, required for mitochondrial fission, showed enhanced cell proliferation on metformin (Fig. 4A) albeit to a less extent than the *efr3* deletion. Thus, indicating that mitochondrial elongation enhances the abilty of cells to proliferate when exposed to metformin. In human cells, the impact of metformin on mitochondrial morphology appears to be cell and context-dependent. It was shown that energy stress and metformin promote mitochondrial fission through activation of AMPK^38,42^. In contrast, metfomin was shown to enhance mitochondrial function, by blocking mitochondrial fission in cardiomyocytes and neurons^40,41^ in part through AMPK activation in high glucose environments^41^. We therefore tested whether fission yeast AMPK mutant cells were resistant or sensitive to metformin. Similar to the *dnm1* deletions, deletion of the AMPK alpha subunit *ssp2* confered some resistance to metformin, but to a lesser extent than the *efr3* deletion (Fig. 4B). This is in agreement with the above finding that the *efr3* deletion confers both AMPK-dependent and AMPK-independent metformin resistance (Fig. 1E).

### Reduced levels of PI4Ps appear to contribute to metformin resistance

Efr3, via the PI4P kinase Stt4, facilitates the generation of PI4Ps at the plasma membrane^22^ (Fig. 1F). PI4Ps act as precursors for the synthesis of PI(4,5)P2s by the PI4P-5-kinase Its3^22^. PI4,5P2s are essential signalling lipids^23^. It has been previously demonstrated that PI4P vesicles are necessary for mitochondrial fission; moreover, elevated levels of PI4P enhance mitochondrial fission, leading to a reduction in mitochondrial length^44,45^. The yeast genome encodes two additional PI4Ks: Pik1, a Golgi-specific PI4 kinase, and Lsb6, localized at the plasma membrane, Golgi, and vacuole membranes. *lsb6* deleted cells have decreased PI4Ps at the plasma membrane^22^, and its deletion also enhances phenotypes of *efr3* deletions^22^. We next examined whether mutants defective in PI4P and PI(4,5)P2 generation: Its3, Pik1 and Lsb6, showed altered response to the anti-proliferative effect of metformin. The *its3-1* mutant, which has reduced PI(4,5)P2 exhibited sensitivity to metformin similar to wild type (Fig. 4C). In contrast, mutants in the two PI4P kinases, Lsb6 and Pik1 showed differing responses to metformin. Whilst the *pik1-11* mutant was more senstive to metformin than wild type cells (Figure S2A), similar to the *dnm1* deletions (Fig. 4A), the *lsb6* deletion confered some resistance to metformin (Fig. 4D). In summary, only reducing PI(4,5)P2 levels as seen in *its3-1* mutants is not sufficient to confer resistance to metformin. By comparison, the *lsb6* and *efr3* deletion mutants, both of which have decreased PI4Ps at the plasma membrane^22^, show resistance to metformin, although *lsb6* to lesser extent than the *efr3* deletion (summarised in Figure S2C). Interestingly, the *efr3* deletion also has reduced PI(4,5)P2 levels^22^, which may in part explain the enhanced metformin resistance in *efr3* mutants. *pik1-11* mutants also have less PI4Ps at the plasma membrane^46^, however, in *pik1.11* mutants PI(4,5)P2 levels are increased which may explain enhanced sensitivity in *pik1.11* mutants (summarised in Figure S2A–C). Overall, reduced PI4P levels appear to correlate with partial metformin resistance.

### Metformin treatment induces mitochondrial fusion in fission yeast

**Fig. 5:**
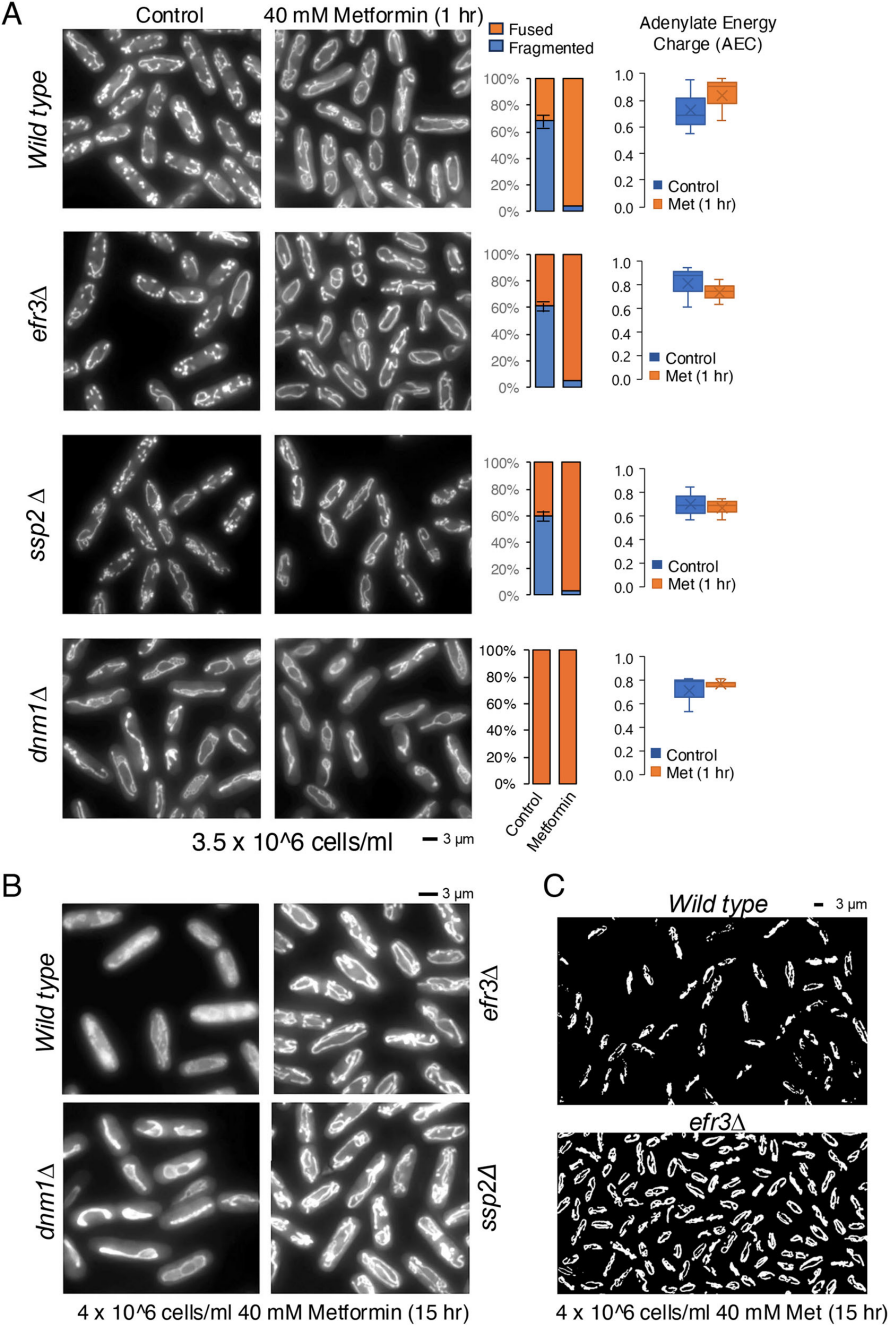
Metformin treatment induces mitochondrial fusion. **A** Live cell imaging of the Cox4-GFP (localised to mitochondria) in mid exponential cultures grown in EMM2 with or without exposure to 40 mM metformin for 1 h. Representative images are shown. Similar results were obtained for three independent biological repeats. Following metformin treatment or non-treated controls, indicated cells were harvested and the adenylate energy charge (AEC) measured by mass spectrometry. **B**, **C** Live cell imaging of the Cox4-GFP (localised to mitochondria) in indicated exponential cultures grown in EMM2 with or without exposure to 40 mM metformin for 15 h. Representative images are shown. Similar results were obtained for three independent biological repeats. Error bars represent mean +/- Standard deviation. **C** Flattened 3D reconstruction of mitochondrial ribbons using Mitochondrial Analyzer Fiji plugin of images from B).

As mentioned, metformin can induce either mitochondrial fission or fusion in human cells, subject to the cell type and environment^38–42^. To explore the effect of 40 mM metformin on mitochondrial morphology in fission yeast, we exposed wild type cells expressing Cox4-GFP (cytochrome c oxidase, complex IV)^47^ to metformin. Firstly, we found that the morphology of mitochondria in steady-state liquid cultures, grown in minimal media EMM2, is dependent on the cell density of the culture. In the early-exponential phase (1 ×10^6 cells/ml), most cells exhibit an elongated mitochondrial morphology (Figure S3A). In contrast, during the mid-exponential phase (3.5 ×10^6 cells/ml), only 60% of cells display mitochondria with an elongated tubular morphology (Fig. 5A).

After one hour of exposure to metformin, over 95% of mitochondria were elongated in the mid-exponential phase. This suggests that metformin triggers mitochondrial fusion in *S. pombe*. Like wild-type cells, approximately 60% of cells in the *efr3* and AMPK alpha (*ssp2*) deletion strains had elongated and tubular mitochondria in steady-state mid-exponential cultures. The addition of metformin also induced mitochondrial fusion in roughly 95% of cells in both mutants (Fig. 5A). In overnight metformin-treated EMM2 cultures that reached the mid-exponential phase after 15 hours, the *efr3*, *ssp2* (AMPK) and *dnm1* (Drp1) deletion strains which show resistance to metformin (Fig. 4A, B), all had elongated mitochondria. However, the mitochondrial filaments in wild-type cells appeared reduced and in part fragmented (Fig. 5B). The 3D reconstruction revealed increased mitochondrial fragmentation in the wild-type strain compared to *efr3* deletion cells (Fig. 5C). Continued measurements of cell densities over the next 6.5 hours demonstrated that all four cultures continued to proliferate although with different generation times (Figure S3B), the *efr3* deletion, most resistant to metformin (Fig. 4), showed the shortest generation time.

We next assessed whether increased fitness of the three deletion mutants when exposed to metformin and compared to wild type, might be related to the cells Adenylate Energy Charge (AEC). AEC quantifies the amount of energy that a cell has at its disposal. It is determined by levels of three key energy molecules present in cells: ATP, ADP and AMP. There were no significant differences between the AECs of wild type and *efr3, ssp2* (AMPKα) and *dnm1* deletion strains (Fig. 5A). Moreover, the addition of metformin for one hour did not significantly change the AEC in any of the strains. Finally, metformin, has been shown to upregulate reactive oxygen species (ROS) in human cells^48^. We considered the possibility that the *efr3, ssp2*, and *dnm1* deletion strains exhibited enhanced fitness when exposed to metformin because of altered levels of reactive oxygen species (ROS) compared to wild type cells. Mutants with altered ROS levels show modified sensitivity to hydrogen-peroxide-generated ROS. However, the ability to form colonies in the presence of hydrogen-peroxide, did not significantly differ in the mutants compared to wild-type cells (data not shown). In summary, acute metformin treatment promotes mitochondria fusion in both wild type and mutant cells but does not significantly change the cellular energy levels. In contrast, in response to prolonged metformin exposure, respiration in the *efr3* deletion is enhanced compared to wild type cells (Fig. 3C), furthermore, less mitochondrial filaments were observed in wild-type cells as they showed increased fragmented (Fig. 5B, C). Hence, mitochondrial fusion might enhance yeast fitness in response to prolonged metformin exposure, also supported by the observation that cells lacking dnm1 (Drp1), with fused mitochondria exhibit resistance to metformin (Fig. 4A).

## Discussion

Prolonged administration of metformin has demonstrated a significant correlation with a reduced incidence of cancer and a positive association with enhanced survival rates across various cancer types^3–8^. Despite this, the full understanding of metformin’s anti-proliferative effects remains elusive and has become a subject of significant interest. In this study, we adopted an unbiased genetic approach, employing the fission yeast model organism, to shed light on cellular mechanisms affected by chronic exposure to metformin. While it is well-established that metformin activates AMPK and independently inhibits mTORC1^13,14^ yeast cells lacking AMPK activity still ceased cell proliferation following exposure to 30 mM metformin (Fig. 1A), however, when exposed to metformin, cells lacking one of the AMPK alpha subunits were somewhat resistant to its anti-proliferative effect when compared to wild type (Fig. 3D). This highlights fission yeast as a model system to explore both AMPK-dependent and non-AMPK-dependent anti-proliferative effects induced by metformin.

Through an unbiased genetic screen for mutants resistant to the anti-proliferative effect of prolonged metformin exposure, we identified several truncation mutants in the Efr3 protein (Fig. 1). Efr3 is a highly conserved plasma membrane protein, it localises Stt4 (PI4K) to the plasma membrane for the generation of PI4Ps^22^. PI4Ps are precursors for the synthesis of PIP2 and PIP3, two essential signalling lipids^23^.

Human EFR3A and EFR3B also regulate KRAS signalling at the plasma membrane in pancreatic cancer cells^25^. Because KRAS signalling is a key driver of cell growth and proliferation, we assessed the possibility that Efr3 was involved in Ras1 signalling in yeast. However, Ras1 localization and signalling are not dependent on yeast Efr3, and efr3 mutants’ resistance to metformin is not modified by mutations in *ras1* (Fig. 2).

In human cells metformin directly inhibits NADH dehydrogenase Complex-I of the electron transport chain^9,10^. The role of Complex-I, which is comprised of 45 subunits, is to transfer electrons from NADH to ubiquinone (coenzyme Q10)^49^ and couple translocation of protons across the mitochondrial membrane. In human cells, MT-MD4 encodes NADH dehydrogenase 4, which is essential for complex 1 activity. The whole mammalian Complex-I is not conserved in fission and budding yeast, in these organisms Ndi1 encodes NADH dehydrogenease (ubiquinone)^50^, where its activity is localised to the mitochondria. Therefore, NADH oxidation by Ndi1 is not directly coupled to proton translocation in yeasts. Notably, Ndi1 from budding yeast *Saccharomyces cerevisiae* can rescue a mitochondria-defective human cell line with a mutation in MT ND4^51^, as transfection with yeast Ndi1 fully restored complex I-dependent respiration and decreased reactive oxygen species levels. Whether metformin directly inhibits yeast Ndi1 is unknown however, importantly our data show that prolonged (but not acute) exposure to metformin also reduced respiration in fission yeast (Fig. 3C).

Inhibition of Complex IV of the electron transport chain by metformin has also been shown in human cells, resulting in an indirect inhibition of mitochondrial glycerol-3-phosphate dehydrogenase GPD2^52,53^. Glycerol-3-phosphate dehydrogenase is part of glycerol metabolism. GPD2 with GPD1 contributes to the glycerol phosphate shuttle, which reoxidizes NADH formed during glycolysis. The disruption of the glycerophosphate shuttle results in an accumulation of reducing equivalents, specifically an elevated ratio of [NADH] to [NAD^+^]. Increased NADH/NAD^+^ ratio is linked to a rise in ROS. Accordingly, metformin was shown to increase NADH/NAD^+^ ratios, upregulate ROS and reduce cancer cell proliferation in human cells^15,48,54^. In cancer cells, metformin also inhibits cell proliferation by suppressing the production of mitochondrial-dependent metabolic intermediates. A mitochondrion “substrate limitation” model of action was proposed for metformin, as citrate required for de novo lipid biosynthesis became depleted when cells were exposed to metformin^16^. Hence, the impact of metformin on mitochondrial function is multifaceted in human cells.

The ability of metformin to block cell proliferation is highly dependent on the environment, which may account for some of the debates around its mechanisms of action. Pyruvate in the culture media was reported to strongly impact the anti-proliferative effect of metformin^15^, this was partly explained by mechanisms that are independent of Complex I, affecting NADH/NAD^+^ ratios. Moreover, decreased ATP/AMP ratios by metformin were reversed by pyruvate in the culture media^15^. In yeast the impact of metformin on cell proliferation is also highly dependent on the environment, we observed that the anti-proliferative effect of metformin is only seen in non-starving exponentially growing cells. In contrast, metformin prolongs the survival and lifespan of starved yeast cells^20^.

Our findings demonstrate that yeast cells lacking *efr3*, required for the generation of PI4Ps at the plasma membrane^22^, display increased fitness and resistance to metabolic stress induced by metformin. Stt4 is the plasma membrane-specific PI4-Kinase localised to the plasma membrane by Efr3. In contrast to Efr3, cells deleted for *stt4* are unviable^55^, suggesting that low levels of PI4Ps at the plasma membrane, as seen in *efr3* deletions (Fig. 1F), are essential for viability. PI4Ps are enriched at both the Golgi apparatus and the plasma membrane^24^. The yeast genome encodes two additional PI4Ks: Pik1, which is an essential Golgi-specific PI4 kinase, and Lsb6, localized at the plasma membrane, Golgi and vacuole membranes. *lsb6* deletion cells have decreased PI4Ps at the plasma membrane^22^, and its deletion enhances phenotypes of *efr3* deletions^22^. Therefore, Lsb6 likely generates low levels of PI4Ps at the plasma membrane in *efr3* deletions. Interestingly, the *lsb6* deletion also confers some resistance to metformin (Fig. 4D) although to a lesser extent than *efr3* mutants. An ability of PI4Ps generated at Golgi membranes to replenish the plasma membranes has been reported in human cells^56^. It has been previously demonstrated that PI4P vesicles derived from the Golgi are necessary for mitochondrial fission^44,45^. If Golgi-derived PI4Ps indeed contribute to the replenishment of the plasma membrane in *efr3* deletion cells, then it is probable that fewer PI4P lipids on Golgi-derived vesicles would be available. This would likely result in mitochondria exhibiting a more fused morphology, as seen in *efr3* deletions during prolonged exposure to metformin (Fig. 5B). Our analysis of several mutants involved in PI4P and PI(4,5)P2 generation (summarized in Figure S2C) shows that mutants with reduced PI4P levels exhibit resistance to metformin, unless PI(4,5)P2 levels are simultaneously increased, as seen in the metformin sensitive *pik1-11* mutant. This accompanying increase of PI(4,5)P2 levels in *pik1-11* mutants may explain why only one of the two Golgi PI4Ks, when mutated (the *lsb6* deletion) confers resistance to metformin, however this will require further investigation. Importantly, our data demonstrate that mutants with reduced PI(4,5)P2 levels only, such as *its3-1*, are as sensitive to metformin as wild type cells. Among all the mutants identified here that confer resistance to metformin, the *efr3* deletion exhibits significantly higher resistance. Whilst, reduced PI4P levels correlated with metformin resistance (Figure S2C), the concurrent reduction in PI(4,5)P2 levels in *efr3* mutants^22^, may explain why only mutations in the *efr3* gene were isolated in our genetic screen. Less stringent selection criteria would facilitate the isolation of mutations in diverse genes in future genetic screens.

Mitochondrial fusion and fission are dynamic processes that play essential roles in mitochondrial quality control, bioenergetics, and overall mitochondrial function. Mitochondrial fusion generally helps maintain mitochondrial health and function by mixing contents between mitochondria and sharing components like proteins, lipids, and DNA^57^. In human cells, the impact of metformin on mitochondrial morphology appears to be cell and context-dependent. It was shown that energy stress and metformin promote mitochondrial fission through activation of AMPK^38,42^, In contrast, metfomin was also shown to enhance mitochondrial function, by blocking mitochodrial fission in cardiomyocytes and neurons^40,41^. In yeast, acute exposure to metformin promoted mitochondrial fusion without affecting cellular energy levels (Fig. 4A). However, only prolonged exposure to metformin reduced respiration (Fig. 3C). Persistent mitochondrial fusion as seen in *efr3* deletion (Fig. 5C) may influence the distribution of key substrates between mitochondria and impact overall cellular fitness. Hence, resistance of *efr3* deletion cells to metformin may in part be related to elongated mitochondrial morphology following prolonged metformin treatments (Fig. 5B), also consistent with the observed metformin resistance and increased fitness in *dnm1* (DRP1) mutants (Fig. 3A) with elongated mitochondria morphology. Recently, in a murine model, metformin was shown to restore cognitive impairment by blocking Drp1-induced mitochondrial fission, thereby increasing cell fitness and reducing mitochondria-generated oxidative stress^40^.

Increased cell fitness in human cells deleted for EFR3 has also been observed. Brain-specific ablation of human EFR3A led to increased neurogenesis by enhancing the survival and maturation of newly generated neurons^58^. This impact on neurogenesis was associated with enhanced brain-derived neurotrophic factor (BDNF) - tropomyosin-related kinase B (TrkB) signalling, including AKT activation, which plays a key role in neural development and maintenance.

In summary, we provide a new perspective on the cellular response and adaptations in relation to the anti-proliferative effects of prolonged exposure to metformin, which operates partly independently of AMPK, a well-established effector protein of metformin. We show that, like human cardiomyocytes and neurons^40,41^, blocking mitochondrial fission enhances cell fitness when exposed to metformin. Hence, the much vaunted anticancer properties of extended metformin treatment may be overridden if mutations affecting mitochondrial morphology arise.

## Materials and Methods

### Yeast cell cultures

Strains used in this study (Table 1). All cultures were grown at 28^o^C and cultured in log phase for 48 h. Cells were inoculated in Yeast extract (YES) based media or Edinburgh minimal media (EMM-N) (Formedium)^59^ supplemented with NH_4_Cl (EMM2)^60^. Exposure of cells to metformin was done by filtering cells and resuspending them in prewarmed media containing metformin at the desired concentration. Metformin was added to YES agar plates at a concentration indicated. All cell proliferation assays (colony forming assays) were performed with cells from early exponential cultures (1.2 ×10^6 cells/ml).

**Table 1:** Strains used in this study

	*Genotype*	Source
JP350	*h*^*+*^	Lab stock
JP1579	*h*^*+*^ *ssp2::ura4+ ura4-D18*	Lab Stock
JP1591	*h*^*+*^ *cbs2::kanMx*	Lab Stock
JP3459	*h- efr3.S60STOP*	This Study
JP3478	*h*^*+*^ *efr3::kanMx ade6-M216 leu1-32 ura4-D18*	Bioneer
JP3515	*h*^*?*^ *efr3::kanMx cbs2::kanMx*	This Study
JP3642	*h*^*?*^ *dnm1::kanMX4 ade6-M216 ura4-D18 leu1-32*	Bioneer
JP3673	*h*^*?*^ *dnm1::kanMx4*	Lab Stock
JP3694	*h*^*-*^ *RasAct-3GFP_leu+ leu1-32*	Sophie Martin YSN3098
JP3695	*h*^*+*^ *leu1-32 GFP-ras1 RasAct_3mCherry_Leu1* +	Sophie Martin YSN3115
JP3696	*h*^*+*^ *ras1.Val17-intURA3* +	NBRP/YGRC
JP3717	*h*^*?*^ *ras1.Val17-intURA3+ efr3::kanMx*	This Study
JP3719	*h*^*?*^ *GFP-Ras1 efr3::kanMx*	This Study
JP3721	*h*^*?*^ *GFP-Ras1*	This Study
JP3722	*h*^*-*^ *efr3::kanMx*	This Study
JP3733	*h*^*+*^ *RasAct-3GFP_leu+ leu1-32 efr3::kanMx*	This Study
JP3768	*h- his5* + *:ppak1:CRIB[gic2aa1-181]-3GFP-terminatorScADH1:kanMX*	Sophie Martin AV2319
JP3780	*h*^*?*^ *efr3::kanMx his5* + *:ppak1:CRIB[gic2aa1-181]-3GFP-terminatorScADH1:kanMX*	This Study
JP3809	*h*^*+*^ *GFP-P4C:leu ade6-M210 ura4-D18 leu1-32*	Kathy Gould KGY19217
JP3865	*h*^*?*^ *GFP-P4C:leu*	This Study
JP3866	*h*^*?*^ *efr3::kanMx GFP-P4C:leu*	This Study
JP3848	*h*^*?*^ *dnm1::kanMx4 efr3::kanMx*	This Study
JP3945	*h*^*?*^ *Cox4-GFP:leu1+ leu1-32 efr3::kanMx*	This Study
JP3970	*h*^*+*^ *cox4-GFP:leu1+ leu1-32*	This Study
JP3917	*h*^*+*^ *cox4-GFP:leu1 ade6-M210 leu1-32 ura4-D18*	PT1650 Phong Tran
JP3984	*h*^*?*^ *cox4-GFP:leu1+ ssp2::ura4+ ura4-D18 leu1-32*	This Study
JP3986	*h*^*?*^ *cox4-GFP:leu1+ dnm1::kanMx4 leu1-32*	This Study
JP4079	*h*^*-*^ *its3-1 ade6-M21x ura4-D18 leu1-32*	Kathy Gould KGY6369
JP4084	*h*^*-*^ *pik1-11:kanMX6 ade6-M21X ura4-D18 leu1-32*	NBRP/YGRC
JP4081	*h*^*+*^ *inp53::kanR ade6-M210 leu1-32 ura4-D18*	Kathy Gould KGY1951-2
JP4038	*h*^*+*^ *lsb6::kanMx ade6-M210 leu1-32 ura4-D18*	Bioneer
JP4086	*h*^*?*^ *its3-1*	This Study
JP4091	*h*^*?*^ *pik1-11:kanMX6*	This Study
JP4088	*h*^*?*^ *inp53::kanR*	This Study
JP4062	*h*^*-*^ *lsb6::kanMx*	This Study

### Genetic Screen

To induce random mutagenesis of the genome, we subjected YES agar plates with both the wild type cells and an AMPK deletion mutant to 150 J UV radiation as described previously^21^. Following overnight growth on YES, the plates were replicated to YES containing 30 mM metformin and resistant colonies selected. Resistant mutants were backcrossed to wild-type cells.

### Semiquantifications of yeast proliferation

Growth of cells was quantified on the 10x dilution spot assay as brightness values using ImageJ and removing background light.

GraphPad Prism 7 was used for data analysis. Unpaired *t* test with Dunnett’s multiple comparisons correction were used for all semi quantifications of growth. 95% confidence of interval was used for calculating significance.

### Fluorescent microscopy

Staining of vacuoles: SynaptoRed™ C2 (Equivalent to FM®4-64) (Cat. # 70021, Biotium) was added to the growth media of cells (1 ×10^6 cells/ml) at a concentration of 1.5 µM for 30 min. Cultures of stained and unstained cells were mixed 1:1 and collected by filtration onto a MF-Millipore™ Membrane Filter, 1.2 µm pore size (Cat. # RAWP04700, Millipore). Cells were resuspended in the original growth media of the FM®4-64 stained cells and subjected to live cell imaging immediately. Images of cells were obtained using a CoolSNAP HQ2 CCD camera. ImageJ was used to measure fluorescence intensity of signals. The total fluorescence intensity (arbitrary units) of GFP fluorescence signals were quantified as: Integrated density of cell—(cell area*mean background brightness of image).

Staining of Cell Wall: FITC Lectin was added to the growth media of cells (1 ×10^6^ cells/ml) at a concentration of 100 µg/ml for 15 min in darkness.

Tip fluorescence was measured as the brightest value on a longitudinal centre line divided by the lowest value in the cytoplasm.

Cox4.GFP imaging: 500 μl unperturbed cultures were spotted directly onto a 35 mm glass-bottom imaging dish (Ibidi, 81218-200) pre-coated with 10 mg/ml Concanavalin A (Sigma, C2010) and imaged immediately using an Eclipse TE2000-E Inverted Microscope (Nikon Instruments) with a Lambda LS xenon-arc lamp (Sutter Instruments). Images were captured using a Cascade II Camera (Photometrics) and Metamorph: Microscopy Automation and Image Analysis Software (Version 7.7.9.0, Molecular Devices) was used to capture z-stacks. 3D reconstruction of mitochondria was carried out using the “Mitochondrial analyzer” Fiji plugin^61^

### Nucleotide measurements

*S. pombe* cells were cultured in EMM2. Cells (1.8×10^6^ cells/ml) were filtered and washed with PBS. Cells were washed off the filter with 400 µl cold 0.5 M perchloric acid. Cells were lysed mechanically with glass beads at 4°C in a FastPrep®-24 homogenizer (MP Biomedicals). Lysates were clarified by centrifugation at 16,000 g for 3 min at 4°C. Clarified extracts were neutralized with 100 µl cold 2.3 M KHCO_3_ and incubated on ice for 5 min. Samples were centrifuged at 16,000 g for 3 min at 4°C. Supernatants were collected and snap-frozen for LC-MS/MS analysis.

Adenine nucleotides were measured as previously described^62^. Briefly, A QTRAP 5500 mass spectrometer (AB Sciex) linked to a Prominence HPLC system (Shimadzu) was controlled and managed with the Analyst 1.7.1 software (AB Sciex). The autosampler was set at 4°C and column oven set at 30°C, which housed a 150 mm (length) × 0.5 mm (inner diameter) Hypercarb 3 µm porous graphitic carbon column (Thermo Fisher Scientific). The LC solvent system comprised of 50 mM triethylammonium bicarbonate buffer (TEAB, Sigma–Aldrich) pH 8.5 in pump A, and acetonitrile with 0.5% trifluoroacetic acid (TFA; Sigma–Aldrich) in pump B. A flow rate of 400 mL.min^−1^ was used throughout a gradient program consisting of 0% B (2 min), 0 to 100% B (10 min), 100% B (3 min), 0% B (2 min). Data was analysed with MultiQuant 3.0.2 software (AB Sciex) using the area under the LC curve. Calibration curves were determined using the Wagner quadratic equation (ln y = a_2_ [ln x]^2^ + a_1_ [ln x] + a_0_) using the peak area of each nucleotide and were required to have a correlation coefficient (R^2^) of >0.99. AEC was calculated from the ratios of [AMP], [ADP] and [ATP] (equation 1):

1 $${\rm{AEC}}=\,\frac{\left[{\rm{ATP}}\right]+\,\frac{1}{2}[{\rm{ADP}}]}{\left[{\rm{ATP}}\right]+\left[{\rm{ADP}}\right]+[{\rm{AMP}}]}$$

### Seahorse analysis

Extracellular Acidification Rate (ECAR) and Oxygen Consumption Rate (OCR) were measured with an XFe96 Extracellular Flux Analyzer (Agilent, California, USA) at 28 degree Celsius according to manufactures instructions. and Seahorse Wave Controller Software 2.6 was used to analyse the data) Seahorse FluxPaks (Cat#103792-100), including sensor cartridges, cell culture microplates and calibrant solution was used. Seahorse XF Cell Mito Stress Test Kit (Cat# 103015-100) was used, final concentrations were 2.5 μM oligomycin, 8.25 μM DCC, 21.5 μM FCCP, 500 nM rotenone and 900 nM antimycin A. Culture plates were coated with Concanavalin A (10 mg/ml) and left to airdry before being used for the assays according to the manufacturer’s instructions. All cultures were early exponential at (1.5 ×10^6 cells/ml) grown in liquid minimal media (EMM2). Optical density OD595, was measured just prior to seeding the cells in the cell culture plate, this was to ensure all wells had an identical number of cells, the metabolic measurements were then performed immediately.

### Statistic analysis

GraphPad Prism software (GraphPad Software Inc, California, USA) was used for all data analysis. 95% confidence of interval was used for calculating significance, *p* value ≤ 0.05 considered as significant. The statistical significance is indicated with asterisk (* *P* ≤ 0.05, ** *P* ≤ 0.01, *** *P* ≤ 0.001, **** *P* ≤ 0.0001).

Unpaired *t* test with Dunnett’s multiple comparisons correction were used for all semiquantifications of growth. Unpaired *t* test was used for PI4P Biosensor and GFP-Ras1 microscopy, and Mann-Whitney test was used for RasAct^GFP^ and CRIB.GFP microscopy. For Seahorse analysis; Unpaired Student’s *t*-test and ANOVA were used to analyse the difference between the means of two and more than two groups, respectively. For nucleotide measurements, statistical significance was calculated using Unpaired Students *t* test.

## Supplementary information

Supplementary material

## Data Availability

No datasets were generated or analysed during the current study.
