# Supplementary material for: Reversal of metformin’s anti-proliferative effect in fission yeast efr3 and dnm1 (DRP1) mutants with elongated mitochondria
Source: NPJ Metab Health Dis. 2025 Feb 21;3:5. doi: 10.1038/s44324-024-00048-9 (PMC11845315; doi:10.1038/s44324-024-00048-9)
Supplement: Supplementary file 1 — Supplementary material [file 44324_2024_48_MOESM1_ESM.pdf]

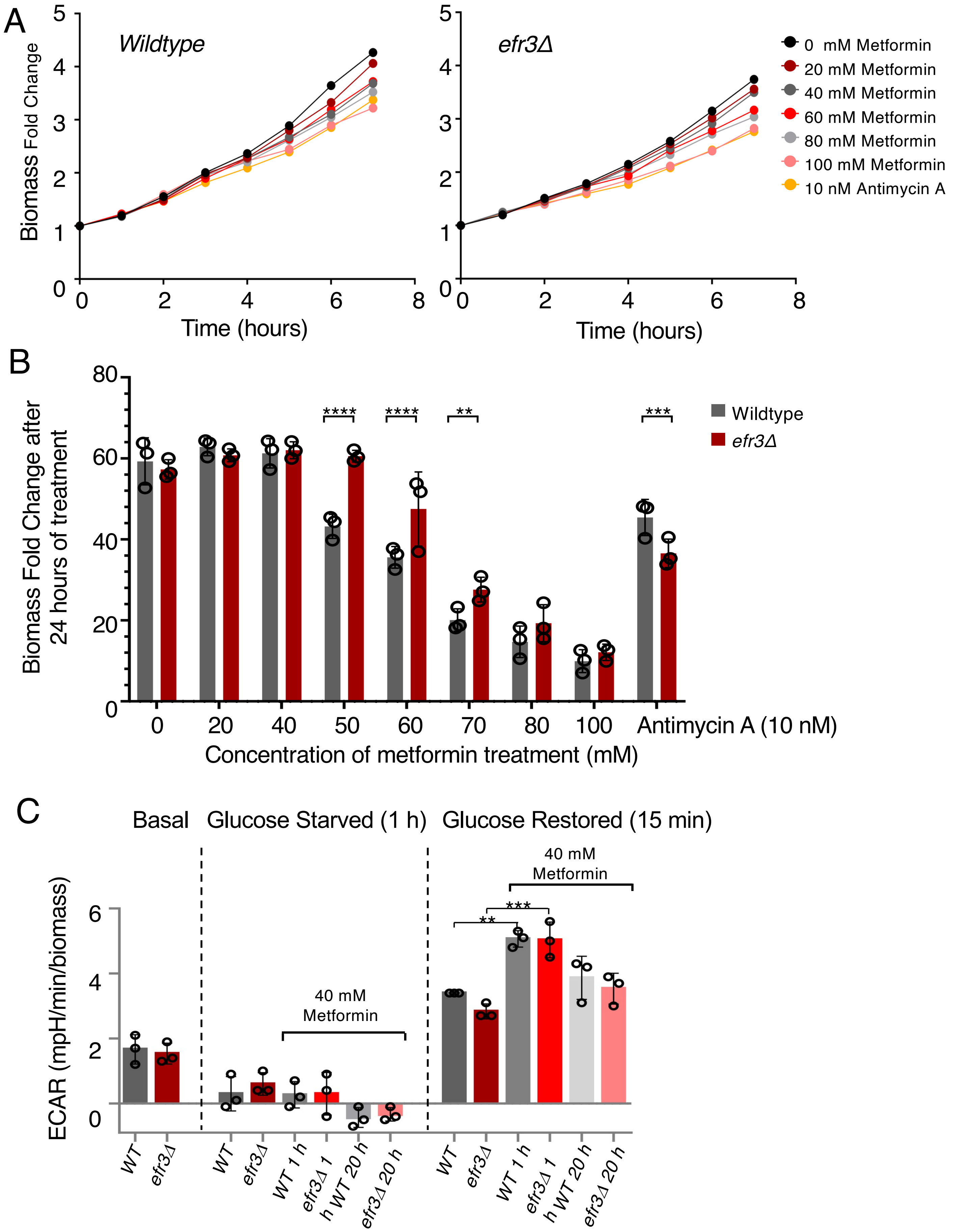


**Figure S1: The effects of acute and prolonged metformin treatment on cell proliferation**

A-B) Metformin or Antimycin A was added to early-exponential cultures (1.5 x $10^6$ cells/ml) grown in liquid minimal media (EMM2) and the increase in biomass ($OD_{595}$) was followed over time. B) The quantification of three independent biological repeats is shown. Error bars represent mean +/- Standard deviation. C) Metabolic glycolytic stress assay of the *efr3* deletion and wild type cells with and without 40mM metformin. Cells were simultaneously starved of glucose for 1 hour and treated with metformin for 1 hour or pretreated with metformin for 19 hours and then starved of glucose for the last hour. The results represent one biological experiment.

A

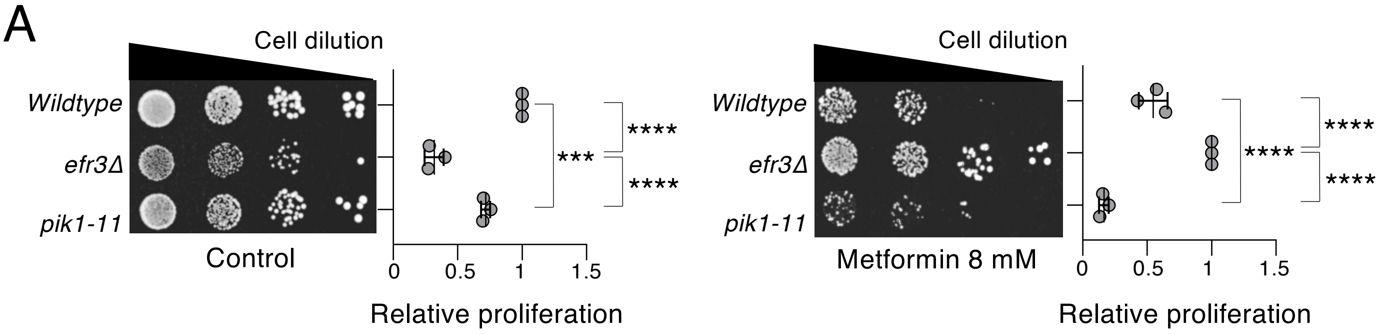


B

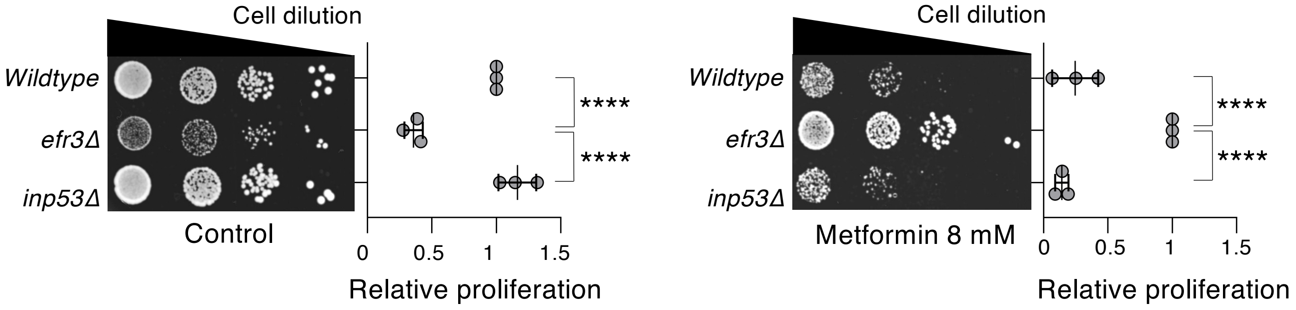


C

| Gene | Mutant allele | Function | Localisation | Response to metformin | PM PI4P | PM PI(4,5)P2 | Reference |
|---|---|---|---|---|---|---|---|
| ***efr3*** | *efr3Δ* | Scaffold for Stt4: A PM specific PI4-kinase | PM | Resistant | Reduced | Reduced | Snider et al. 2018 |
| ***lsb6*** | *lsb6Δ* | PI4-kinase | PM, Golgi and Vacuoles | Resistant | Reduced | Normal | Snider et al. 2018, Willet et al. 2023 |
| ***its3*** | *its3-1* | PI4P-5-kinase | PM | Sensitive | Normal/small increase | Reduced | Snider et al. 2018 |
| ***pik1*** | *pik1-11* | PI4-kinase | Golgi | Sensitive (more than wt) | Reduced* | Increased* | Willet et al. 2023 |
| ***inp53*** | *inp53Δ* | PI4,5P-5-phosphatase | Division site, cytosol | Sensitive | Not assessed | Increased | Snider et al. 2018 |

* Has increased Its3 on PM (Snider et al. 2018)

**Figure S2: Two mutants with reduced PI4P levels exhibit resistance to metformin**
A-B) Growth characteristics of indicated strains on rich YES media with or without 8mM metformin. The quantification of three independent biological repeats is shown. Error bars represent mean +/- Standard deviation. C) Summary of growth characteristics of mutants involved with PI4P and PI4,5P generation following exposure to metformin. Plasma membrane = PM.

A

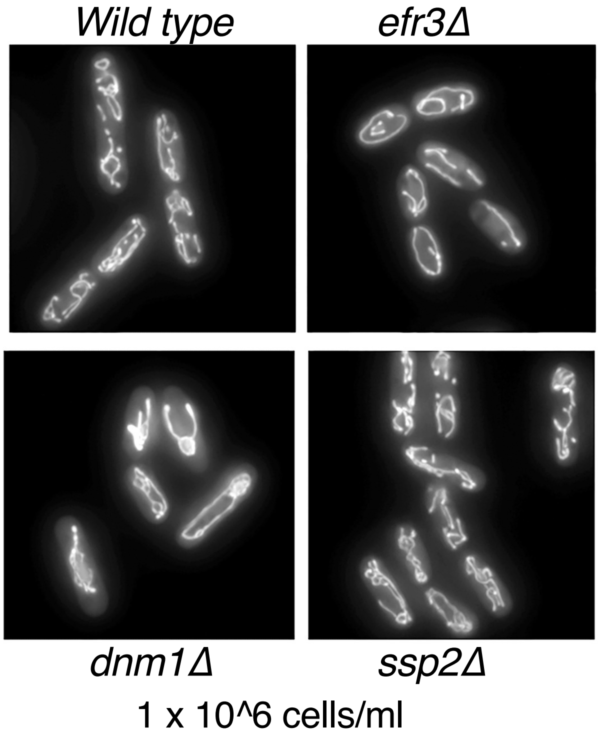


B

| Genotype | 15 hr cells/ml | 19 hr cells/ml | 21.5 hr cells/ml | Generation Time (hours:mins) (15hr-21.5hr metformin) | Generation Time Control media |
|---|---|---|---|---|---|
| *wt* | 3.9 x 10^6 | 5.5 x 10^6 | 7.1 x 10^6 | 8:01 | 3:45 |
| *efr3Δ* | 3.5 x 10^6 | 6.8 x 10^6 | 9.8 x 10^6 | 4.45 | 4:10 |
| *ssp2Δ* | 4.0 x 10^6 | 6.4 x 10^6 | 8.3 x 10^6 | 6.23 | 3:55 |
| *dnm1Δ* | 3.7 x 10^6 | 6.0 x 10^6 | 7.5 x 10^6 | 6.28 | 4:20 |

**Figure S3: Early exponential cultures have tubular mitochondria.**
A) Live cell imaging of the Cox4-GFP (localised to mitochondria) in early exponential cultures grown in minimal media EMM2. Representative images are shown. Similar results were obtained for three independent biological repeats. B) Generation times of the same cells used in figure 5B following a further 6.5 h of proliferation.
